# Structure-Dependent Stability of Lipid-Based Polymer Amphiphiles Inserted on Erythrocytes

**DOI:** 10.3390/membranes11080572

**Published:** 2021-07-29

**Authors:** Chunsong Yu, Myunggi An, Meng Li, Charles Manke, Haipeng Liu

**Affiliations:** 1 Department of Chemical Engineering and Materials Science, Wayne State University, Detroit, MI 48202, USA; chunsong.yu@wayne.edu (C.Y.); myunggi.an@wayne.edu (M.A.); fx9138@wayne.edu (M.L.); charles.manke@wayne.edu (C.M.); 2 Tumor Biology and Microenvironment Program, Barbara Ann Karmanos Cancer Institute, Detroit, MI 48201, USA

**Keywords:** erythrocytes, membranes, amphiphiles, lipid

## Abstract

Cell-based therapies have the potential to transform the treatment of many diseases. One of the key challenges relating to cell therapies is to modify the cell surface with molecules to modulate cell functions such as targeting, adhesion, migration, and cell–cell interactions, or to deliver drug cargos. Noncovalent insertion of lipid-based amphiphilic molecules on the cell surface is a rapid and nontoxic approach for modifying cells with a variety of bioactive molecules without affecting the cellular functions and viability. A wide variety of lipid amphiphiles, including proteins/peptides, carbohydrates, oligonucleotides, drugs, and synthetic polymers have been designed to spontaneously anchor on the plasma membranes. These molecules typically contain a functional component, a spacer, and a long chain diacyl lipid. Though these molecular constructs appeared to be stably tethered on cell surfaces both in vitro and in vivo under static situations, their stability under mechanical stress (e.g., in the blood flow) remains unclear. Using diacyl lipid-polyethylene glycol (lipo-PEG) conjugates as model amphiphiles, here we report the effect of molecular structures on the amphiphile stability on cell surface under mechanical stress. We analyzed the retention kinetics of lipo-PEGs on erythrocytes in vitro and in vivo and found that under mechanical stress, both the molecular structures of lipid and the PEG spacer have a profound effect on the membrane retention of membrane-anchored amphiphiles. Our findings highlight the importance of molecular design on the dynamic stability of membrane-anchored amphiphiles.

## 1. Introduction

Cell-based therapy has been an active area of research for many years, and is a rapidly expanding field in treating many pathologies, including cancer, neurological diseases, and autoimmune disorders [1,2,3,4,5]. Because the molecular landscape of plasma membrane determines all interactions of the cell with its environment, therapeutic cells are often engineered at the surface to regulate cell functions, such as cell survival, adhesion, migration, targeting, and cell–cell interactions, or to enable targeted drug transport [6,7]. Cell surface engineering utilizes engineering, chemistry, and biology techniques to enable modification of cell surface molecules with targeting ligands, biological functionalities, nanoparticles, or synthetic drugs. However, the complex and dynamic nature of the cell surface poses a major challenge for efficient modification without physically compromising vital cell functions such as viability, adhesion, migration, and immune recognition [7].

To date, many approaches for cell surface engineering have been reported [6,7]. For example, genetic engineering is a versatile and powerful methodology to express or suppress specific cell surface molecules. Chimeric antigen receptor-modified T (CAR-T) cells, which are genetically engineered to express CAR targeting tumor-specific molecules, have demonstrated great success in the treatment of cancer [8]. Several therapies based on CAR-T cells are now available for hematological cancers [9]. However, technical difficulties (e.g., genetic engineering is not amenable to all cell types, and a large portion of the therapeutic materials (e.g., synthetic drugs, imaging tags, polymers, nanoparticles, DNA) cannot be introduced genetically) and safety concerns have complicated its applications in the clinical setting [10]. By contrast, covalent chemical modification is another recently developed approach that can be used to attach a broad range of functional molecules to the cell surface [6]. This approach targets cell surface functional groups including amines, thiols, and carboxyls present in proteins, carbonhydrates, or lipids. A major drawback of covalent functionalization is the possible interference with the function of native proteins and cells. Moreover, the degree of modification is believed to be difficult to control. Cell surface functionalization by lipid-based amphiphiles which are capable of exogenously inserting into the plasma membrane is an attractive alternative approach which can overcome the above limitations [11,12,13,14]. In this method, hydrophobic interaction is the main driving force which anchors the amphiphiles on the cell membrane. To facilitate the spontaneous membrane insertion, these amphiphiles are designed with a functional group of interest conjugated to a suitable hydrophobic anchor (e.g., lipid or steroid) via a solubility promoting polymer (e.g., polyethylene glycol, PEG). This approach is simple and noninvasive, allowing rapid and uniform surface functionalization of a wide variety of molecules without compromising the cell viability and functions. Using lipid-conjugated PEGs, investigation of the insertion and dissociation of lipid-conjugated PEGs with different lipid lengths was conducted [11,15,16]. It was found that although a shorter alkyl chain lipid (1,2-dimyristoyl-sn-glycerol-3-phosphatidylethanolamine, DMPE, 14 carbons) exhibited rapid membrane incorporation, it was also rapidly dissociated in a cell culture context. Membrane insertion of a longer alkyl chain lipid (1,2-distearoyl-sn-glycerol-3-phosphatidylethanolamin, DSPE, 18 carbons) was slow, a comparable density can be achieved when high concentrations were used. As expected, DSPE-PEG exhibited prolonged surface retention after initial membrane insertion. The exact mechanisms of how these membrane-anchored polymer dissociate have not been fully characterized. However, cell membrane dynamics, albumin-binding, and competition via neighboring cells might jointly contribute to the kinetic loss over time. Similar observations were obtained for lipid-modified amphiphilic oligonucleotides, where both the length of the hydrophobic tails and oligos affect the efficiency of insertion as well as retention [15,16].

Although the static stability of these lipid-polymers anchored on the cell surface has been evaluated, the stability under mechanical shear stress (e.g., in the blood flow) remains uncharacterized. Since many cells used in cell-based therapies need to enter the blood circulation, it is important to evaluate the retention of membrane-anchored amphiphiles under shear stress and hemodynamic forces [7]. In this study, we analyzed the effect of molecular structures of lipid-PEG amphiphiles on their kinetic stability on cell surface under mechanical shear stress. Using erythrocytes as a cellular model, we report in vitro and in vivo data that provide insight into 1) the kinetics of lipid-PEG surface retention as a function of lipid and PEG chain length; and 2) the key biochemical interactions affecting surface persistence in a complex mechanical and biochemical environment.

## 2. Results and Discussion

**The hydrophobicity of the lipid and the length of PEG control the stability of lipo-PEG on the cell surface under static conditions**. The spontaneous insertion of lipid-based amphiphiles into the cell surface membranes is well-documented in previous studies [11,12,14]. Our previous data revealed a complex three-way equilibrium when lipid-based polymer amphiphiles were exposed to biological fluids [12,17,18]. The amphiphilic lipo-polymers can self-assemble into spherical micelles in aqueous buffer. However, due to the relatively short hydrophobic moiety, these micelles are thermodynamically unstable under physicochemical conditions. In the presence of serum albumin and cells, lipo-polymer micelles dissemble themselves and predominantly bind to albumin protein or insert into plasma membrane. Interestingly, the equilibrium between albumin binding state and membrane insertion state is controlled by the chain length of both lipid and polymer block [17]. While amphiphiles with short diacyl lipids and a short polymer block are rapidly inserted into the cell membrane, the spontaneous insertion was less efficient and slower for amphiphiles with long lipid tails and long polymer blocks [17]. Additionally, the high affinity between albumin protein and long-chain lipids also shifts the equilibrium toward the albumin-binding state, especially at high albumin concentrations [17,18,19].

**Figure 1:**
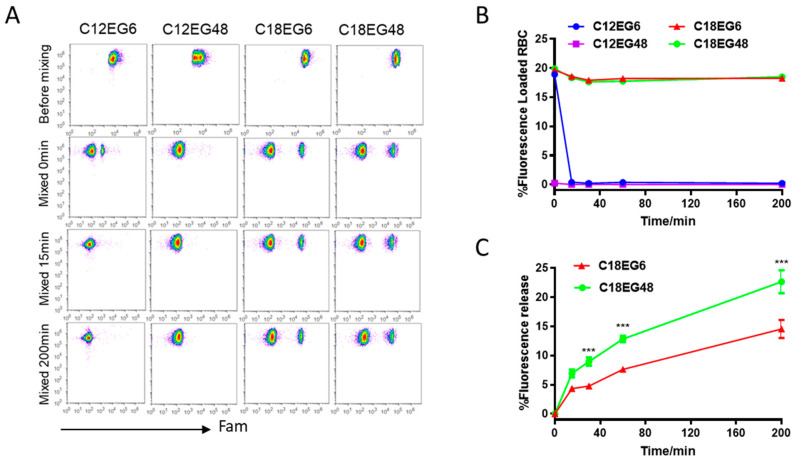
Kinetic stability of lipo-PEGs with varying length of lipid and PEG on RBC surface. (**A**–**C**), mouse RBCs were loaded with different Fam-labeled lipo-PEGs and cocultured with fleshly isolated mouse blood. The decay of fluorescence on RBC surface was quantified by flow cytometry. (**A**) Dot plot of RBC at different time points. (**B**,**C**) Time dependent Fam-positive frequencies (**B**) and relative mean fluorescence intensities (**C**) of RBCs. Each sample was assayed with three replicates. Error bars represent the SEM. Data show the mean values ± SEM. *, *p* < 0.05; **, *p* < 0.01; ***, *p* < 0.001; ****, *p* < 0.0001; ns, not significant.

We envisioned that varying the amphiphile’s structures would affect the hydrophobic/hydrophilic balance, and subsequently affect the stability of lipo-polymers anchored on plasma membrane. To test this hypothesis, we synthesized a panel of lipid-modified polyethylene glycol (PEG) with varying lengths of lipid and PEG linker (Figure S1). To minimize the cell turnover and membrane internalization, we chose the red blood cells (RBCs), as mature erythrocytes have reduced endocytosis and can circulate in the bloodstream for up to 120 days [20,21]. We first loaded dye-labeled lipo-PEGs on RBCs. To reduce the competitive binding from serum proteins, RBCs were separated from serum and resuspended in PBS buffer. To compensate for the differences in loading efficiency and kinetics for different lipo-PEGs, we used high concentrations of lipo-PEGs to achieve a high initial loading efficiency on RBC surface. Briefly, 5 µM of Fam (carboxyfluorescein)-labeled lipo-PEGs were incubated with RBCs in PBS buffer at 37 °C for 1 h and the unloaded lipo-PEGs were subsequently removed by repeated centrifugation and washing. Using these procedures, relatively uniform membrane insertions were achieved for all four lipo-PEGs, showing a clear Fam-positive cell population on the flow dot plot (Figure 1A). However, the fluorescence intensity of RBCs loaded with long chain lipids (C18) was almost 10 times higher than RBCs loaded with short chain lipids (C12), regardless of their PEG lengths, indicating that a significantly higher density and stable insertion was achieved by amphiphiles with long chain lipids (Figure 1A).

To mimic the complex biological environment after parenteral injection, lipo-PEGs loaded RBCs were mixed with freshly isolated whole blood at a volume ratio of 1:10 and subsequently incubated at 37 °C. The retention of lipo-polymers on RBC surface was quantified by flow cytometry over time. Similar to previous studies [16,17,18,19,20], amphiphilic polymers with short lipid tails (12 carbons) rapidly dissociated from the RBC surface when mixed with whole blood. Fifteen min after incubation, no cell populations with fluorescent population can be detectable in both short lipid short PEG (C12EG6)- and short lipid long PEG (C12EG48)-treated RBCs, suggesting a rapid and complete release of short lipid tail-polymers (C12PEG) from RBC surface when mixed with blood (Figure 1A,B). It appeared that the dissociation of C12EG48 from the RBC surface was extremely rapid, as the Fam-positive population disappeared right after mixing with blood. By contrast, the frequency of Fam-positive cells remains constant in RBCs loaded with long lipid (C18) for more than 3 h, although their fluorescence intensity gradually decreased (Figure 1C). These results suggest that a sufficient hydrophobicity is required to firmly anchor the payload on RBC surface in the presence of whole blood. Interestingly, we also observed the amphiphiles with a long PEG block is released at a faster rate than shorter PEG. As shown in Figure 1C, short PEG with six EG units remains attached on cells longer than long PEG with 48 units. It is worth pointing out that none of the PEG modified with C18 lipids causes hemolysis in RBCs (Figure S2). Thus, the reductions in fluorescence intensity were caused by the release of polymers from initial cell surface into blood, most likely by the increased interaction between high molecular weight lipid-PEG with serum protein.

**Figure 2:**
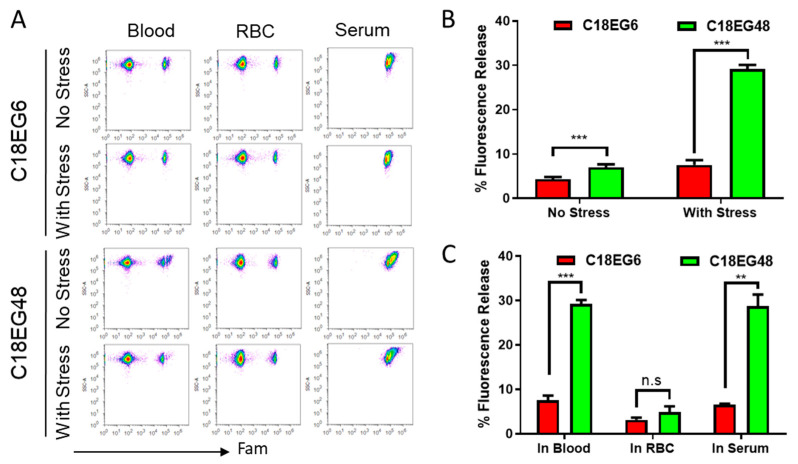
Shear stress-induced release of lipo-PEGs on RBC surface. C18-PEGs-loaded RBCs were mixed with whole blood, or unloaded RBCs, or serum, and shear stress was applied for 15 min. Cells were assayed by flow cytometer. Flow dot plot (**A**) and percentage of release (**B**,**C**) of lipo-PEGs from RBCs w/wo shear stress. Plot (**B**) shows the percentage of fluorescence release of lipo-PEGs in blood with or without shear stress. Plot (**C**) shows the fluorescence release after 15 min shearing at 10 Pa from lipo-PEGs loaded RBCs in each of the suspension media. Each sample was assayed with three replicates. Error bars represent the SEM. Data show the mean values ± SEM. *, *p* < 0.05; **, *p* < 0.01; ***, *p* < 0.001; ****, *p* < 0.0001; ns, not significant.

**The effect of shear stress on the stability of membrane-anchored lipid polymers.** Having demonstrated the structure-based stability of lipid-polymers under static conditions, we set out to evaluate the impact of mechanical stress on the lipid-polymer loaded on the RBCs’ surface. Unlike static cell culture conditions, cells are exposed to mechanical stresses at various levels of the circulatory system under physiological conditions [22]. Thus, the stability of membrane anchored lipid polymers under mechanical stress might be different from that under static conditions. To test this hypothesis, lipid-PEGs-loaded RBCs were subjected to in vitro shear flow conditions. Parenterally injected drugs are exposed to three major types of fluid shear stresses, caused by blood flow, interstitial fluid flow and lymphatic fluid flow. Estimates of wall shear stress in these fluids have been made, with the highest stress observed in the circulating blood [22]. Studies have found that physiological mean shear stress depends on vascular locations, with levels ranging from 1.0–2.0 Pa in arteries to 0.1–0.6 Pa in veins [23]. However, the value can rise to well above 10 Pa, in vessels with high flow rates and small diameters [24]. Lipo-PEGs-loaded RBCs were subjected to high shear stress in vitro, using a Rheometrics ARES rheometer with parallel plate geometry, operated to provide constant shear stress. Because short lipid-PEGs rapidly released from RBC surface in co-culture settings, we focus on the long lipid-PEGs. As shown in Figure 2A,B, under mechanical stress, the amphiphiles were released significantly faster than static conditions. After applying 10 Pa of shear stress for 15 min, 7.5% C18EG6 and 29.2% C18EG48 were released from RBC surfaces, as compared to 4.3% and 6.9% under static conditions. In both dynamic and static situations, long EG polymer was released at a higher rate than short EG polymer, suggesting that the length of polymer block is an important factor that controls the membrane’s stability after insertion.

In our experiment, the amphiphiles-loaded RBCs were mixed with freshly isolated whole blood. It is possible that the release was caused by competitive lipid insertion from unloaded RBCs or the binding with serum proteins. To investigate the role of different blood components in the observed release, the amphiphiles-loaded RBCs were sheared separately after mixing with the same amount of purified RBCs or blood serum. Shearing lipo-PEG-loaded RBCs with empty RBCs resulted in 3.1% and 4.8% reductions in the mean fluorescence for C18EG6 and C18EG48, respectively. Both values were significantly less than those with RBCs sheared in whole blood (Figure 2C). In contrast, RBCs sheared with mouse serum resulted in similar levels of fluorescence reduction as compared to whole blood (Figure 2C). Together, these data demonstrated that blood serum was the dominant factor responsible for the shear-induced release.

To simulate the physiological conditions of blood flow-induced shear stress, amphiphiles loaded RBCs were mixed with whole blood and pumped through a capillary tube (0.1 mm in diameter). The pressure and flow rate were controlled by regulating the pumping speed so that 10 Pa of shear stress was achieved. Similar release profiles were observed by this extracorporeal model (Figure S3). These two in vitro models demonstrated that mechanical stress accelerates the release of lipid-based amphiphiles on RBC surface.

**Figure 3:**
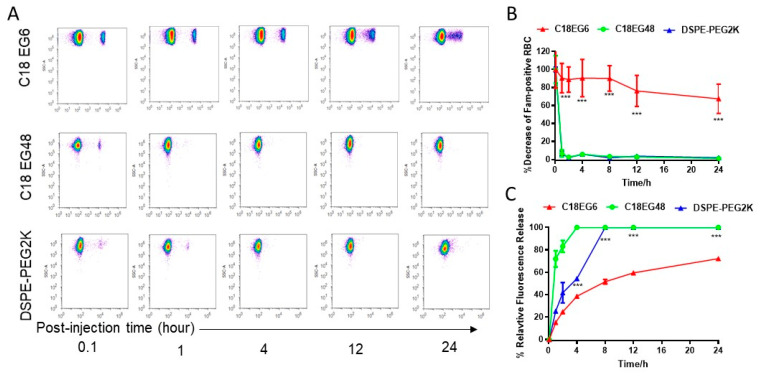
In vivo release kinetics of C18EG6, C18EG48, and DSPE-PEG2K loaded RBCs. (**A**) Representative flow cytometry dot plots. (**B**,**C**) The relative frequencies of Fam-positive RBCs (**B**) and mean fluorescence intensities (**C**) at different time points after injection. Each time point was assayed with three replicates. Error bars represent the SEM. *, *p* < 0.05; **, *p* < 0.01; ***, *p* < 0.001; ****, *p* < 0.0001; ns, not significant. Statistical analyses were made by comparing C18EG6 and C18EG48 groups.

**Shear stress affects the in vivo stability of membrane-anchored lipid-polymers in vivo.** In vivo, drugs are exposed to different levels of blood flow-induced shear stress, consistent with differences in flow rates/velocity, vessel diameters, and disease states. To investigate the in vivo stability of amphiphile loaded on RBCs, C18PEGs loaded RBCs were injected intravenously into mice. Blood samples were analyzed by flow cytometry at different time points. As shown in Figure 3, the release rate of long PEG (C18EG48) loaded on RBCs was significantly faster when compared to that at static culture. Under static conditions, the frequencies of C18PEG-positive RBCs remained constant over 3 h of incubation (Figure 1). However, 1 h after injection, the frequency of Fam-positive RBCs dropped to an undetectable level for C18EG48 (Figure 3A). In contrast, C18EG6 exhibited a significantly prolonged retention on RBC surface after i.v. injection, as demonstrated by a clearly detectable and slow decay of the Fam-positive RBC frequencies and mean fluorescence intensities after injection (Figure 3 and Figure S4). Although the release rates of lipo-PEGs loaded on RBCs in vivo were faster than those in cell culture (static) conditions, the impact of shear stress on the release rate appeared to be more significant for long PEG than that for short PEG (Figure 1C vs. Figure 3C).

**Cationic lipid-PEG conjugate significantly enhances the membrane stability in vivo.** The fast dissociation rate of C18EG48 from the RBC surface in vivo raises the question of how to improve the stability of similar payloads with long PEG linkers. For synthesis purposes, the C18EG series we used in the above studies were synthesized by using a DNA synthesizer with phosphorothioate linkages. Because the extracellular components of plasma membrane are negatively charged, it is possible that the highly negatively charged C18EG48 promotes the dissociation after insertion. However, a commercial PEG amphiphile (DSPE-PEG2K) with C18 diacyl lipid and ~45 EG units showed fast dissociation from RBC surface in vivo (Figure 3), suggesting that electrostatic repulsion is not a dominant factor. Pegylation of liposomes with compatible length of PEG has been a known procedure to improve the liposome stability in vivo, especially in blood circulation [25]. Studies have also consistently showed that long PEG chains positively correlate with significantly more release from the liposome membranes [26]. On the other hand, fluorescent lipophilic membrane dyes (e.g., long-chain dicarbocyanine) are widely used to label plasma membrane for long-term cell tracking in vivo. For long-term dye retention on targeting cells, these dyes share some common design characteristics, such as fluorophore directly conjugating to the long aliphatic tail to ensure strong hydrophobic interactions with surround lipids, and a positive charge to provide additional electrostatic interaction. However, due to the relatively short hydrophilic fluorescent head, in salt-containing buffers, these dyes rapidly form micelles or aggregate and require special diluent for maximal staining.

**Figure 4:**
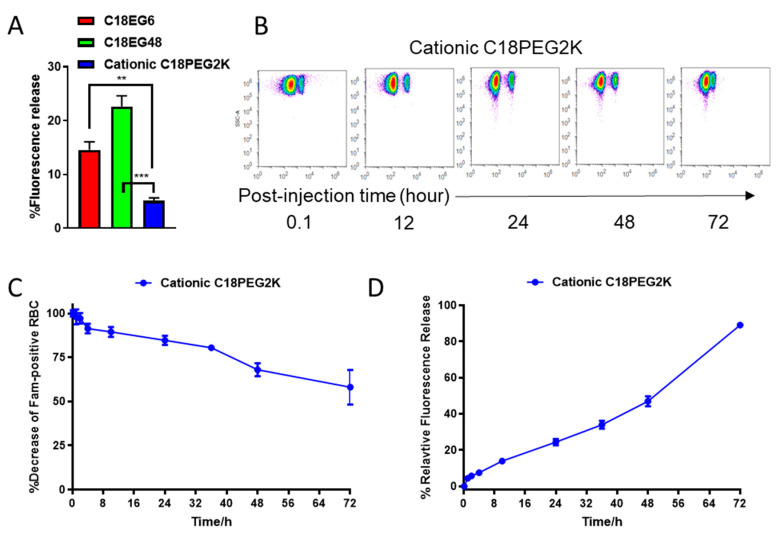
Cationic lipid prolongs the retention time of long PEG amphiphiles in vitro and in vivo. (**A**), Percentages of release cationic C18PEG2K after in vitro culture with whole blood. (**B**–**D**) Representative flow cytometry dot plots (**B**) and relative frequencies of Fam-positive RBCs (**C**) and mean fluorescence intensities (**D**) at different time points after injection. Each time point was assayed with three replicates. Error bars represent the SEM. *, *p* < 0.05; **, *p* < 0.01; ***, *p* < 0.001; ****, *p* < 0.0001; ns, not significant.

In many cases, a long linker is preferred for the retention of both ligand and cellular functions. However, the fast dissociation rate of C18-EG48 from the RBC surface in vivo suggests that hydrophobic interaction alone is insufficient for the retention of payload with long linker. Since plasma membranes are negatively charged, we postulate that a positively charged lipid tail would provide charge–charge interaction in addition to hydrophobic interaction, and can prolong the retention time of long EG polymers on plasma membrane. We synthesized a cationic C18 lipid and conjugated it with fluorescein-labeled long EG (Figure S5). The retention of cationic PEG2K (~45 EG) was significantly prolonged in static conditions (Figure 4A and Figure S6), as compared to both C18EG6 and C18EG48 cocultured with freshly isolated blood for 3 h. Importantly, in vivo, cationic lipid significantly improved the retention kinetics of long EG as compared to the original phospholipid, characterized by a 40-fold improvement in retention half-life on RBCs. Twenty-four hours after injection, 24.3% of the cationic C18EG48 polymer is released from RBCs, as compared to 100% of anionic C18EG48. Forty-fold improvement in the retention half-life on RBCs was observed. There were considerable amounts of cargo remaining on the RBCs even after 72 h in circulation. We concluded that a cationic lipid design facilitates the long-term retention of amphiphilic cargo anchored on RBCs during circulation.

## 3. Conclusions

Surface modification of cells to regulate cell behaviors and functions is essential in cell-based therapies, and is frequently used for drug delivery, cell and organ transplantation, and tissue engineering. To facilitate the application of lipid-based amphiphiles as an alternative and standard approach for fast, non-toxic modification of the cell surface, it is of major importance to understand the molecular designs that affect the anchoring stability of these amphiphiles in vivo. Our findings in this study indicate that the structures of lipid-based amphiphiles play an important role which dominates the stability under shear-stress. While short chain lipid (C12)-modified PEG rapidly released from RBC surface, long chain lipid (C18)-modified PEG significantly prolonged the release kinetics. Additionally, the length of PEG block also impacts the anchoring stability post insertion, with higher stability associated with shorter PEG. A similar trend can be observed when cells were subjected shear stress in vitro and in vivo. In a situation where long PEG block is preferred, our data indicate that additional electrostatic interaction might be employed, as positively charged diacyl lipid significantly improved the stability of long PEG in vivo. It is worth pointing out that the stability data were obtained by using RBC as a model cell, which has limited endocytic activities. The dynamic behaviors of the cell membrane of other cell types involve a wide variety of membrane trafficking processes. Additionally, depending on the types and their locations, cells are exposed to distinct fluid components and shear stresses. For example, the composition of both interstitial fluids and lymphatic fluid differs considerably from the blood. Thus, the stability of lipid-based amphiphiles on the cells in these structures might need further investigation. Together, using a panel of synthetic lipid-based amphiphiles, the membrane-anchoring stability on cell surface was followed and quantified, in both static and shear flow conditions. Further investigations will shed light on optimal molecular structures that maximize the stability in vivo.

## Data Availability

The data presented in this study are contained within the article and supplementary material.

## Supplementary Materials

The following are available online at https://www.mdpi.com/article/10.3390/membranes11080572/s1, Figure S1: Structures of C12 and C18 lipid-modified PEG amphiphiles. Figure S2: RBCs hemolysis induced by insertion of amphiphilic cargoes. Figure S3: In vitro pumping-generated shear stress induced release of lipo (EG)n-inserted on RBC. Figure S4: Confocal images of lipo-PEG inserted RBC before and post injection. Figure S5: Structures of commercial DSPE-PEG2K and cationic C18PEG2K amphiphiles. Figure S6: Cationic C18PEG2K greatly improves the in vitro stability when loaded on RBC surface.
