# Supplementary material for: Structure-Dependent Stability of Lipid-Based Polymer Amphiphiles Inserted on Erythrocytes
Source: Membranes (Basel). 2021 Jul 29;11(8):572. doi: 10.3390/membranes11080572 (PMC8402226; doi:10.3390/membranes11080572)
Supplement: Supplementary file 1 [file membranes-11-00572-s001.zip › membranes-1302109-supplementary.pdf]

membranes

Supplementary information

Structure-Dependent Stability of Lipid-Based Polymer Amphiphiles Inserted on Erythrocytes

Chunsong Yu [1], Myunggi An [1], Meng Li [1], Charles Manke [1] and Haipeng Liu [1,2,*]

[1] Department of Chemical Engineering and Materials Science, Wayne State University, Detroit, MI 48202, USA; chunsong.yu@wayne.edu (C.Y.); myunggi.an@wayne.edu (M.A.); fx9138@wayne.edu (M.L.); charles.manke@wayne.edu (C.M.)
[2] Tumor Biology and Microenvironment Program, Barbara Ann Karmanos Cancer Institute, Detroit, MI 48201, USA;
* Correspondence: to: haipeng.liu@wayne.edu

Table of contents

S1. Supplementary Materials and Methods

S1.1 Materials

S1.2 Synthesis of diacyl lipid phosphoramidite

S1.3 Synthesis and purification of Fluorescein labeled lipo-$(EG)_n$

S1.4 Synthesis of cationic lipid

S1.5 Synthesis and purification of FITC labeled cationic lipo PEG

S1.6 Synthesis of DSPE-PEG-FITC

S1.7 Insertion of amphiphilic cargo on RBC *ex vivo*

S1.8 *In vivo* kinetics study

S1.9 The exertion of shear stress on RBC

S1.10 The hemolysis of RBCs

S2. Supplementary Results

`

S1. Supplementary Materials and Methods

S1.1 Materials

Chemicals and reagents. DMT hexaethyloxy glycol phosphoramidite and all reagents used on ABS 394 DNA synthesizer were purchased form Glenres or Chemgenes and used following manufacturer's instructions. Vybrant DiD Cell-Labeling Solution was purchased from Thermo Scientific. 3′-FAM resin was purchased from Allele Biotechnology. DSPE-PEG-NH_2 was bought from Laysan Bio Inc. NHS-FITC and NHS-PEG-FITC 2000 were purchased from Nanocs Inc. All other reagents were purchased from Sigma-Aldrich except where otherwise noted.

Animals and cells. Animals were housed in the United States Department of Agriculture (USDA)-inspected Wayne State University animal facility under federal, state, local and NIH guidelines for animal care. Female C57BL/6 mice (5-8 weeks) were obtained from the Jackson Laboratory. Cells were cultured in complete medium (MEM, 10% fetal bovine serum (Greiner Bio-one), 100 U/mL penicillin G sodium and 100 μg/mL streptomycin (Pen/Strep).

S1.2 Synthesis of diacyl lipid phosphoramidite

The C18 diacyl lipid phosphoramidite was synthesized as previously described[1-2]. A solution of stearoyl chloride (6.789 g, 22.41 mmol) in 1,2-dichloroethane (50 mL) was added dropwise to a solution of 1,3-diamino-2-dydroxypropane (1.0 g, 11.10 mmol) in the presence of 1,2-dichloroethane (100 mL) and triethylamine (2.896 g, 22.41 mmol). The reaction mixture was stirred for 2 h at 25 °C and then heated at 70 °C overnight. The reaction mixture was then cooled to 25 °C, filtered, and the solid was sequentially washed with CH_2Cl_2, CH_3OH, 5% $NaHCO_3$, and diethyl ether. The product was dried under vacuum to give the intermediate product as a white solid (yield: 90%). 1H NMR (300 MHz, $CDCl_3$, ppm): δ 6.3 (m, 2H), 3.8 (m, 1H), 3.4–3.2 (m, 4H), 2.2 (t, 4H), 1.6

`

(m, 4H), 1.3–1.2 (m, 60H), 0.9 (t, 6H). The intermediate product (5.8 g, 9.31 mmol) and N,N-diisopropylethylamine (DIPEA, 4.2 mL, 18.62 mmol) was then suspended in anhydrous CH_2Cl_2 (100 mL). The mixture was cooled on an ice bath and 2-cyanoethyl N,N- diisopropylchlorophosphoramidite (8.6 mL, 0.47 mmol) was added dropwise under dry nitrogen. After stirring at 25 °C for 1 h, the solution was heated to 60 °C for 90 min. The solution was washed with 5% $NaHCO_3$ and brine, dried over Na_2SO_4, and concentrated under vacuum. The final product was isolated by precipitation from cold acetone to afford 4 g (55% yield) lipid phosphoramidite as a white solid. 1H NMR (300 MHz, $CDCl_3$): δ 6.4 (m, 2H), 3.9 (m, 2H), 3.8 (m, 2H), 3.6 (m, 2H),
3.0–2.9 (m, 2H), 2.6 (t, 2H), 2.2 (m, 4H), 1.6 (m, 6H), 1.3– 1.2 (m, 72H), 0.9 (t, 6H). 31P NMR ($CDCl_3$): 154 ppm. The C12 diacyl lipid phosphoramidite was synthesized following the same procedures above except that the final product was purified by silica gel column chromatography.

S1.3 Synthesis and purification of Fluorescein labeled lipo-$(EG)_n$

All anionic lipo-$(EG)_n$ were synthesized using an ABI 394 synthesizer on a 1.0 micromole scale. DMT-hexaethyloxy glycol phosphoramidites was conjugated as a 'base' initiated from 3′-(6-Fluorescein) labeled controlled pore glass. All lipophilic phosphoramidites were conjugated as a final 'base' on the 5′ end of sequence in the DNA synthesizer. After synthesis, lipo-$(EG)_n$ were cleaved from the solid support and deprotected, purified by HPLC using a reverse phase C4 column (BioBasic-4, 200mm X 4.6mm, Thermo Scientific), 100mM triethylamine-acetic acid buffer (TEAA, pH7.5)-Methanol (0-15min, 50-100%) as an eluent. Fluorescein labeled lipo-$(EG)_n$ typically eluted at 14 min detected by wavelength of 480nm. All other lipo-PEG amphiphiles were purified by HPLC using the same eluting method.

S1.4 Synthesis of cationic lipid

To a 50 mL round-bottom flask, 0.6 g (3.744 mmol) of NBoc-ethylenediamine and 3.744 g (11.232 mmol) of octadecyl bromide was taken in ethyl acetate (12 mL) and refluxed in the presence of anhydrous potassium carbonate (2.069 g; 14.976 mmol) for 24 h. After completion, the mixture was diluted to 100 mL ethyl acetate, and washed with water (2 X 100 mL), dried over anhydrous magnesium sulfate and filtered. Ethyl acetate was removed by rotary evaporation. The residue was purified by silica gel column chromatography to afford 0.5g

`

intermediate product (10% ethyl acetate in hexane, v/v, as the eluent).MS(LC-MS) calculated: m/z 664.68; found: 665.67. To a 25mL round-bottom flask, the intermediate product and 0.3g methyl iodide was taken in 10mL acetone and refluxed for 24h. The reaction mixture was concentrated under reduced pressure and the residue was washed twice with 50mL hexane by centrifuge for 10min at maximum speed to yield 0.3g white powder. MS(LC-MS) calculated: m/z 679.71; found: 679.55. The white powder obtained (0.3g, 0.44mmol) was dissolved in 6mL of anhydrous dichloromethane (DCM) and 2mL trifluoroacetic acid (TFA) was added. The reaction mixture was stirred at room temperature for 24h. After completion, the reaction mixture was mostly concentrated by rotary evaporator and vacuumed by oil pump overnight without further purification. Afterwards, 10mL NaOH (1N) and 10mL dichloromethane were added the unpurified product and the biphasic mixture was stirred at room temperature (RT) for 2h. The organic layer was washed with water (20mL x 3), dried over magnesium sulfate and filtered. The solvent in the filtrate was completely removed by rotary evaporator and then vacuumed oil pump overnight to afford 0.15g final product. MS(LC-MS) calculated: m/z 579.66; found: 579.56

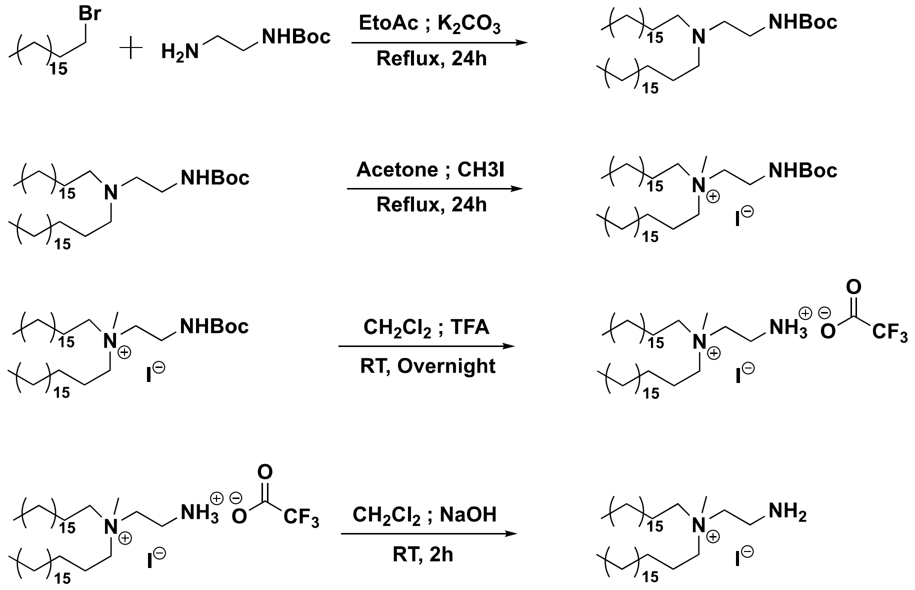

S1.4 Scheme of synthesis of cationic lipid

S1.5 Synthesis and purification of FITC labeled cationic lipo PEG.

6 mg (10.51 μmol) of cationic lipid and 13 mg (4.2 μmol) of NHS-PEG-FITC 2000 were dissolved in 1 mL dimethylformamide (DMF) and the reaction mixture was stirred at RT overnight in the presence of 1 μL of

`

triethylamine (TEA). After completion, the mixture was purified by HPLC using aforementioned method.

S1.6 Synthesis of DSPE-PEG-FITC

5.2mg of DSPE-PEG-NH2 2000 and 1.7mg of NHS-FITC was dissolved in 1mL dimethylformamide (DMF) and the reaction mixture was stirred at RT overnight in the presence of 1μL of triethylamine (TEA). After completion, the mixture was purified by HPLC.

S1.7 Insertion of amphiphilic cargo on RBC *ex vivo*

Fresh blood was collected from Balb/c mice. 10 μL whole blood was washed twice with PBS (1x) and resuspended in 100 μL PBS. Then fluorescein labeled lipo PEG was added into RBC suspension at a final concentration of 5 μM and incubated at 37°C for 1 h. Afterwards, samples were washed with PBS (1X) twice to remove unbound lipo PEG, and the labeled cells was further incubated with whole blood, plasma serum (original concentration in whole blood) or pure RBC suspension (original cell density in whole blood). At designated time points, a small aliquot of the mixture was taken out and then assayed by flow cytometry.

S1.8 *In vivo* kinetics study

RBC from 100 μL whole blood was treated with fluorescein labeled lipo PEG as described previously and resuspended in 150 μL PBS (1X). If necessary, RBC was further treated with DiD dye after amphiphilic cargo insertion. Balb/c mice were i.v. injected with 150 μL treated RBC suspension at tail. Blood samples were collected at tail tip for flow cytometry analysis at designated time points.

S1.9 The exertion of shear stress on RBC

RBC was treated as previously described. 5 μL treated RBC was mixed with 100 μL whole blood or RBC suspension or plasm serum (diluted to physiological concentration with 1X PBS). After mixing, samples were then loaded onto ARES rheometer plate and sheared at 3000 1/s for 15 mins at room temperature. When shearing was completed, 1 μL fresh sample was collected immediately for flow cytometry analysis. Besides shear stress

`

exerted by rheometer, a syringe pump with a flow rate of 0.30 mL/min was set to shear samples mixed with whole blood for 12 mins.

S1.10 The hemolysis of RBCs

Freshly collected blood was washed with PBS (1X) and then was resuspended in PBS (1X) with 50 times dilution. Each well 200 μL of RBCs suspension was incubated at 37°C for 1 h with 5 μM amphiphilic cargoes, 1% Triton-X 100 (v/v) or without treatment in a round-bottomed 96-well plate. For each group, samples were loaded in quadruplicate. After incubation, the plate was centrifuged for 5 min at 500 x g to pellet intact RBCs. 100 μL of supernatant from each well was transferred into a flat-bottomed 96-well plate. The absorbance of hemoglobin was measured by UV-vis plate reader at 540 nm. After background subtraction, the hemolysis percentage of each group was normalized to positive control Triton-X 100 group which represented 100% hemolysis.

S1.11 Statistical analysis

All plots represent mean values and error bars represent the standard error of the mean (SEM). Comparisons of the mean values of two groups were performed using unpaired Student's t-tests. One-way analysis of variance (ANOVA), followed by a Bonferroni post-test, was used to compare >2 groups. *, $p<0.05$; **, $p<0.01$; ***, $p<0.001$; ns, not significant unless otherwise indicated. Statistical analysis was performed using GraphPad Prism 6 software (San Diego, CA).

`

S2. Supplementary Results

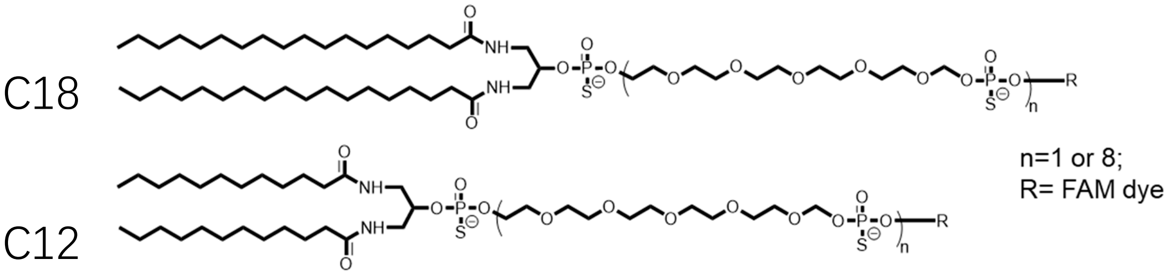

Figure S1. Structures of C12 and C18 lipid-modified PEG amphiphiles.

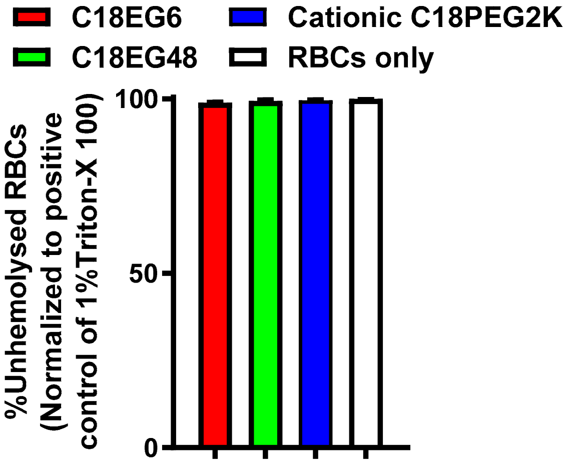

Figure S2. RBCs hemolysis induced by insertion of amphiphilic cargoes. 200 µL RBCs suspension was incubated with 5 µM amphiphilic cargoes or 1% Triton-X 100 or nothing for 1h at 37°C. The supernatant was assayed to detect the absorbance by RBC hemoglobin at 540 nm. Triton-X 100 served as positive control, while no treatment group was negative control. Each specimen was assayed with four replicates. Error bars represent the SEM.

`

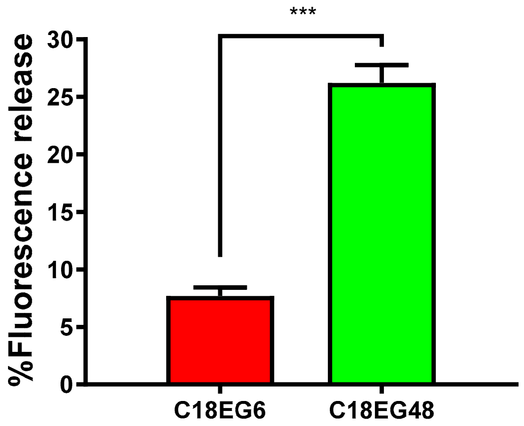

Figure S3. ***In vitro*** **pumping-generated shear stress induced release of lipo (EG)n-inserted on RBC.** 5 μL of Fam labeled lipo PEG-inserted RBC was mixed with 200 μL whole blood, diluted in 600 μL PBS and loaded into 1 ml BD syringe. Sample was then pumped with a syringe pump with a flow rate of 0.30 mL/min. After pumping, 10 μL of blood sample was collected for flow cytometry analysis. The bar graph represent the percentage of relative MFI decrease of C18 lipid conjugated $(EG)_6$ or $(EG)_{48}$ inserted RBC in blood samples after pumping. Each specimen was assayed with three replicates. Error bars represent the SEM. NS, no significance*p<0.05; **p<0.005; ***p<0.001; ****p<0.0001, tow-tail unpaired Student *t* test.

`

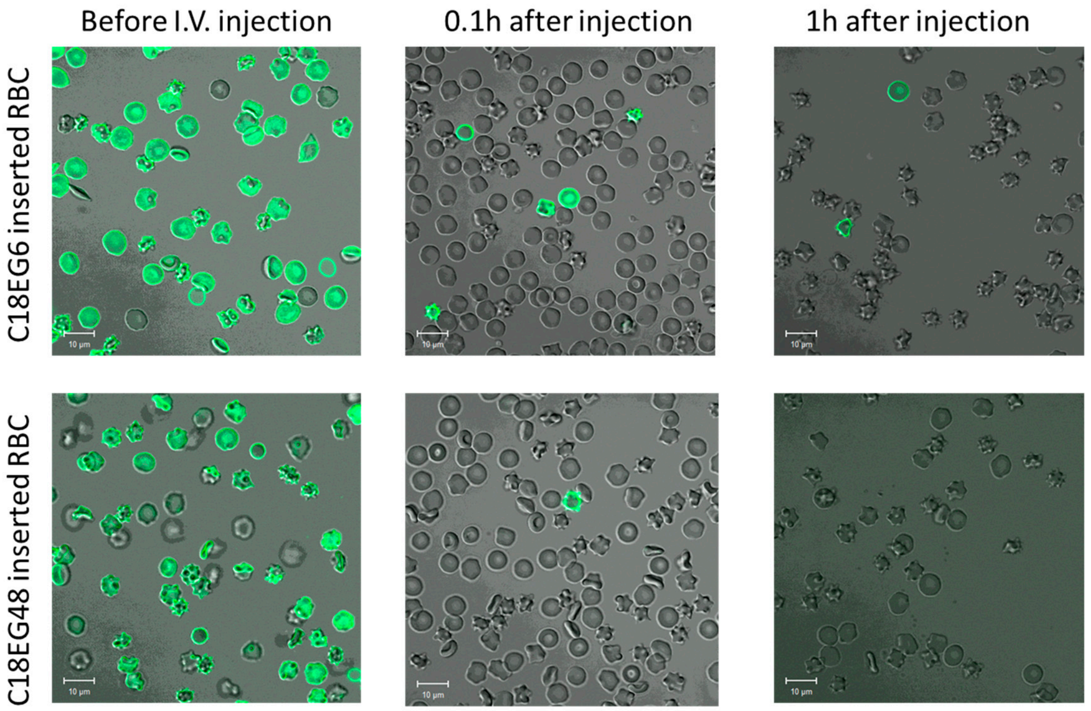

Figure S4. Confocal images of lipo-PEG inserted RBC before and post injection. Red blood cells were treated as previously described and i.v. injected, blood samples were collected at designated time point and imaged by Carl Zeiss LSM 800 confocal microscope.

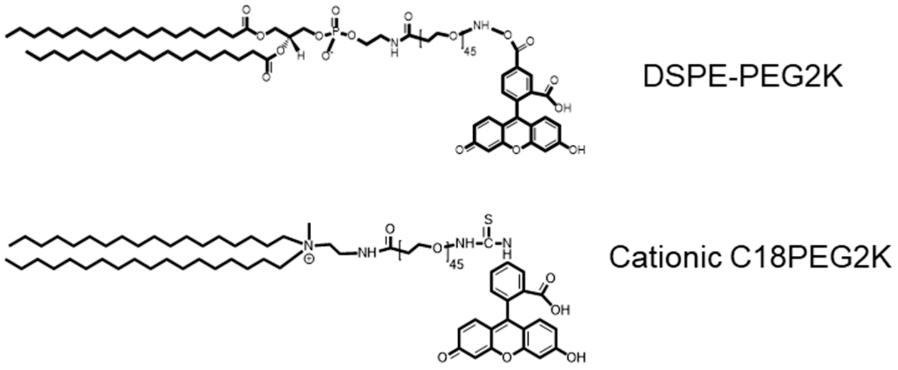

Figure S5. Structures of commercial DSPE-PEG2K and cationic C18PEG2K amphiphiles.

`

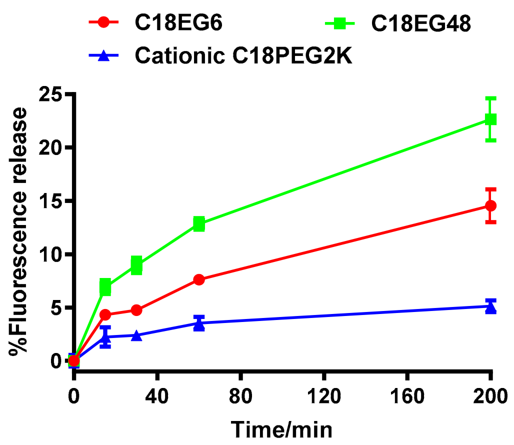

Figure S6. Cationic C18PEG2K greatly improves the in vitro stability when loaded on RBC surface. 5 μL Fam-labeled lipo PEG-inserted RBC was mixed with 100 μL whole blood and then incubated at 37°C. The relative release percentage of C18 lipid conjugated PEG at different time points was quantified by measuring the mean fluorescence intensities of each sample.

1. Yu, C.; An, M.; Li, M.; Liu, H., Immunostimulatory Properties of Lipid Modified CpG Oligonucleotides. *Molecular pharmaceutics* **2017,** *14* (8), 2815-2823.

2. Liu, H.; Moynihan, K. D.; Zheng, Y.; Szeto, G. L.; Li, A. V.; Huang, B.; Van Egeren, D. S.; Park, C.; Irvine, D. J., Structure-based programming of lymph-node targeting in molecular vaccines. *Nature* **2014,** *507* (7493), 519-522.
